# Precision Imaging in Neurodegeneration: The Superiority of Diffusion Tensor Imaging Over Conventional MRI in Differentiating Parkinson’s Disease From Atypical Parkinsonian Syndromes

**DOI:** 10.7759/cureus.68933

**Published:** 2024-09-08

**Authors:** Jasvant Ram Ananthasayanam, Prashanth Anandan, Arunkumar Mohanakrishnan, Keerthi Charitha

**Affiliations:** 1 Radiodiagnosis, Saveetha Medical College, Saveetha Institute of Medical and Technical Sciences, Saveetha University, Chennai, IND; 2 Medical Imaging Technology, Saveetha Medical College, Saveetha Institute of Medical and Technical Sciences, Saveetha university, Chennai, IND

**Keywords:** atypical parkinsonian syndromes (aps), diffusion tensor imaging (dti), mri, parkinson's disease (pd), progressive supranuclear palsy - richardson syndrome (rs)

## Abstract

Background

Parkinson's disease (PD) is a progressive neurodegenerative disorder characterized by the degeneration of dopaminergic neurons in the substantia nigra, leading to motor and non-motor symptoms. Atypical parkinsonian syndromes (APS), including progressive supranuclear palsy (PSP) and essential tremor (ET), present with overlapping clinical features, making differential diagnosis challenging. Conventional MRI has limitations in distinguishing PD from APS, necessitating advanced imaging techniques like diffusion tensor imaging (DTI) for more accurate diagnosis.

Objectives

This retrospective study aimed to evaluate the diagnostic accuracy of DTI in diagnosing PD and APS, particularly assessing its ability to differentiate these conditions from each other compared to conventional MRI. Additionally, the study sought to determine if DTI could diagnose PD in cases where conventional MRI results were normal, thereby highlighting the potential role of DTI in enhancing diagnostic precision in neurodegenerative disorders.

Methodology

The study included 30 patients with clinically diagnosed PD or APS who underwent both conventional MRI and DTI. Data were collected retrospectively. Imaging was performed using a Philips Multiva 1.5-Tesla MRI scanner (Philips, Amsterdam, Netherlands). DTI sequences were analyzed for fractional anisotropy (FA) values in the substantia nigra, superior cerebellar peduncle, middle cerebellar peduncle, transverse pontine fibers, and dentate nucleus. The FA values were compared with established normal values, and the findings from DTI were correlated with clinical diagnoses and conventional MRI results.

Results

Among the 30 patients, 53.3% were clinically diagnosed with PD and 46.7% with APS, including PSP and ET. Conventional MRI findings were normal in 46.7% of cases, indicating its limitations in detecting early or subtle changes in neurodegenerative disorders. In contrast, DTI identified abnormalities in 83.3% of cases, demonstrating its superior diagnostic sensitivity. DTI detected significant FA value reductions in the substantia nigra in PD patients (mean FA: 0.440), which is consistent with the degeneration of dopaminergic neurons characteristic of PD. In PSP patients, the superior cerebellar peduncle showed marked FA reductions (mean FA: 0.523), correlating with the clinical features of PSP, such as bradykinesia and postural instability. ET was identified by reduced FA values in the superior cerebellar peduncle and dentate nucleus, distinguishing it from other forms of parkinsonism. DTI was particularly effective in cases where conventional MRI results were inconclusive or normal, identifying early-stage PD and differentiating it from APS with greater accuracy. The study demonstrated a sensitivity of 95.8% and specificity of 93.8% for DTI in differentiating PD from APS compared to conventional MRI.

Conclusion

This study highlights DTI as a superior imaging modality for the early diagnosis and differentiation of parkinsonian disorders, particularly when conventional MRI results are inconclusive. DTI’s ability to detect significant microstructural changes in specific brain regions, evidenced by FA value reductions, enhances diagnostic accuracy. Incorporating DTI into routine clinical practice is essential for accurate differentiation between PD and APS, facilitating better patient management.

## Introduction

Parkinson’s disease (PD) is recognized as one of the most common and debilitating neurodegenerative disorders, characterized by the progressive degeneration of dopaminergic neurons in the substantia nigra [1]. This degeneration leads to a spectrum of motor symptoms, including bradykinesia, rigidity, resting tremor, and postural instability, as well as non-motor symptoms such as cognitive decline, mood disorders, and autonomic dysfunction. The diagnosis of PD is predominantly clinical, relying on the identification of these characteristic symptoms. However, the clinical presentation of PD often overlaps with that of atypical parkinsonian syndromes (APS), a group of disorders that include conditions such as progressive supranuclear palsy (PSP), multiple system atrophy (MSA), and corticobasal degeneration (CBD). These conditions, collectively termed Parkinson’s plus syndromes (PPS), share several features with PD but have distinct underlying pathologies and prognoses [1]. The differentiation between PD and APS is crucial not only because of the differences in disease progression and management strategies but also due to the implications for patient prognosis. Conventional MRI has long been a cornerstone in the evaluation of neurodegenerative diseases, providing valuable information on structural brain changes [2]. However, its utility in distinguishing between PD and APS is limited. Conventional MRI sequences, such as T2-weighted and proton density imaging, may reveal some degree of atrophy or signal changes in the advanced stages of these diseases, but they often fail to detect the subtle microstructural changes that occur in the early stages, which are critical for early diagnosis and intervention [3].

In recent years, diffusion tensor imaging (DTI), an advanced MRI technique, has gained prominence in neuroimaging due to its ability to provide detailed insights into the integrity of white matter tracts. DTI operates by measuring the diffusion of water molecules along these tracts, producing quantitative metrics such as fractional anisotropy (FA) that reflect the microstructural organization of the brain’s white matter. In the context of neurodegenerative diseases, DTI offers a unique advantage by detecting early microstructural changes that are not visible on conventional MRI [4]. For instance, in PD, DTI has demonstrated the ability to identify reduced FA values in the substantia nigra, correlating with the loss of dopaminergic neurons. Similarly, in APS such as PSP, DTI can detect abnormalities in the superior cerebellar peduncle and other brain regions involved in the disease, thereby aiding in its differentiation from PD. The clinical implications of these advancements are profound. Early and accurate differentiation between PD and APS can significantly influence treatment decisions, patient counseling, and the overall management of the disease [5]. For example, while PD patients may benefit from dopaminergic therapies and surgical interventions like deep brain stimulation, these treatments are often less effective in APS, where other therapeutic strategies may be more appropriate.

This study is designed to rigorously evaluate the diagnostic performance of DTI in comparison to conventional MRI in differentiating PD from APS. By focusing on the analysis of FA values in key brain regions, including the substantia nigra, superior cerebellar peduncle, middle cerebellar peduncle, transverse pontine fibers, and dentate nucleus, this research aims to establish DTI as a superior imaging modality with higher sensitivity and specificity for early diagnosis [6]. The study leverages a retrospective approach, ensuring a comprehensive analysis of both newly acquired and previously collected imaging data, thereby providing robust evidence for the clinical utility of DTI. The results of this study are expected to have significant implications for the future of neuroimaging in neurodegenerative diseases. By demonstrating the superiority of DTI over conventional MRI, the study could pave the way for its broader adoption in clinical practice, ultimately improving diagnostic accuracy, guiding treatment decisions, and enhancing the quality of life for patients affected by parkinsonian syndromes [7].

## Materials and methods

This retrospective study, conducted in the Department of Radiology, aimed to compare the diagnostic efficacy of DTI with conventional MRI in differentiating PD from APS. The study included 30 patients aged 30 years and above who were either clinically diagnosed or highly suspected of having PD or APS. Patients with contraindications for MRI, such as those with metallic implants or severe claustrophobia, were excluded from the study. The research was carried out over six months. As per our institutional guidelines, retrospective studies utilizing de-identified data do not mandate formal ethics approval. Informed consent was obtained from all participants.

MRI was performed using the Philips Multiva 1.5-Tesla MRI scanner (Philips, Amsterdam, Netherlands). The imaging protocol included conventional MRI sequences (T1-weighted, T2-weighted, diffusion-weighted imaging (DWI), fluid-attenuated inversion recovery vascular (FLAIR), fast field echo (FFE)) as well as advanced DTI sequences (3D T1-weighted, DTI iso media, dual turbo spin echo (TSE), susceptibility-weighted imaging (SWI)). The total scan time for each patient was approximately 30 minutes. For image acquisition, patients were positioned supine with the head first, using a sense head coil for immobilization. FA maps were generated from the DTI images, with regions of interest (ROIs) placed in key brain regions, including the substantia nigra, superior cerebellar peduncle, middle cerebellar peduncle, transverse pontine fibers, and dentate nucleus. The caudal segment of the substantia nigra was emphasized due to its higher specificity in diagnosing PD. The FA values obtained were then compared with established normal values. For each ROI, two independent measurements of FA values were taken, and the average FA value was recorded. This process ensured consistency and minimized variability in the measurements.

Statistical analysis was performed using both descriptive and inferential statistical methods. The primary analysis involved the use of student’s paired t-tests to compare FA values between different groups, with a p-value of less than 0.05 considered statistically significant, indicating a meaningful difference between the groups. To assess the reliability of the FA measurements, intraclass correlation coefficient (ICC) testing was performed. An ICC value greater than 0.75 was used to indicate good reliability of the measurements across different brain regions. Descriptive statistics, such as means, medians, and standard deviations, were used to summarize the FA values across the study population. To ensure the robustness of the findings, the study also examined the sensitivity and specificity of DTI in comparison to conventional MRI, with results cross-referenced against confirmatory clinical diagnoses. Radiologists were blinded to both clinical and imaging diagnoses to prevent and reduce bias. The comparative analysis demonstrated that DTI provided superior sensitivity and specificity in distinguishing PD from APS, supporting the integration of DTI into routine diagnostic protocols for parkinsonian disorders.

## Results

A total of 30 cases were included in the study, with the ages of participants ranging from 31 to 90 years. The majority of cases, 12 (40%), belonged to the age group between 61 and 70 years. Among the 30 cases, 21 cases (70%) were male and nine cases (30%) were female, indicating a higher prevalence of the condition among males compared to females (Table 1).

**Table 1:** Age and gender distribution of study participants

Category	Frequency	Percentage (%)
Age distribution		
31 - 40 years	1	3.3%
41 - 50 years	2	6.7%
51 - 60 years	8	26.7%
61 - 70 years	12	40.0%
71 - 80 years	6	20.0%
81 - 90 years	1	3.3%
Total	30	100%
Gender distribution		
Male	21	70%
Female	9	30%
Total	30	100%

Among the 30 patients, a majority, 11 cases (36.7%), were diagnosed with PD. Essential tremor (ET) and atypical parkinsonian disorder (APD) each accounted for five cases, that is 16.6% of the patients. PD with associates were 16 patients (53.3%). PSP was observed in three cases, that is 10% of the patients, while drug-induced parkinsonism, vascular parkinsonism, and dementia each constituted two cases, that is 6.7% of the total. APD with associates were 14 patients (46.7%). The patient group comprised 21 males (70%) and nine females (30%), indicating a higher prevalence of these conditions in males (Table 2).

**Table 2:** Distribution based on clinical diagnosis

Clinical Diagnosis	Number of Patients (Male)	Number of Patients (Female)	Total Number of Patients	Percentage (%)
Parkinson's disease	9	2	11	36.7%
Essential tremor	4	1	5	16.6%
Atypical parkinsonian disorder	4	1	5	16.6%
Progressive supranuclear palsy	2	1	3	10.0%
Drug-induced parkinsonism	1	1	2	6.7%
Vascular parkinsonism	1	1	2	6.7%
Dementia	1	1	2	6.7%
Total	21	9	30	100%

Representative cases

Case 1

**Figure 1:**
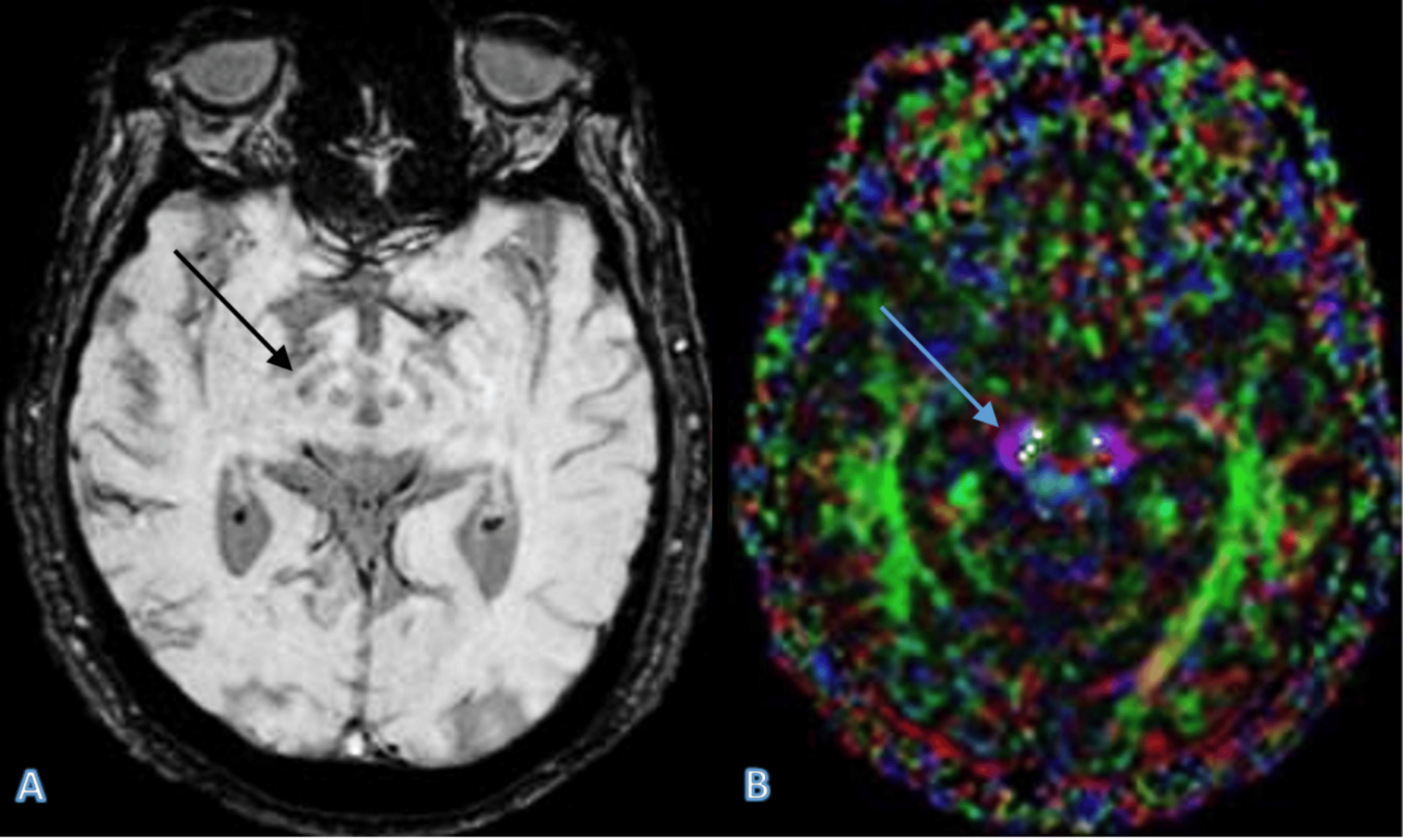
The SWI image (A) displays a clear and well-defined swallow tail sign in the substantia nigra (indicated by the arrow), which is typically lost in Parkinson’s disease. The DTI image (B) shows intact white matter tracts with FA values consistent with normal findings, further supporting the absence of Parkinson’s disease. Arrows in both images highlight the key areas of interest. SWI: Susceptibility-weighted imaging; DTI: Diffusion tensor imaging; FA: Fractional anisotropy

A 65-year-old male patient presented with clinical symptoms consistent with idiopathic PD. Despite the clinical suspicion, both SWI and DTI confirmed the absence of significant degeneration in the substantia nigra, ruling out PD in this case. The normal "swallow tail" sign and FA values suggested that other causes for the patient’s symptoms should be considered (Figure 1).

Case 2

**Figure 2:**
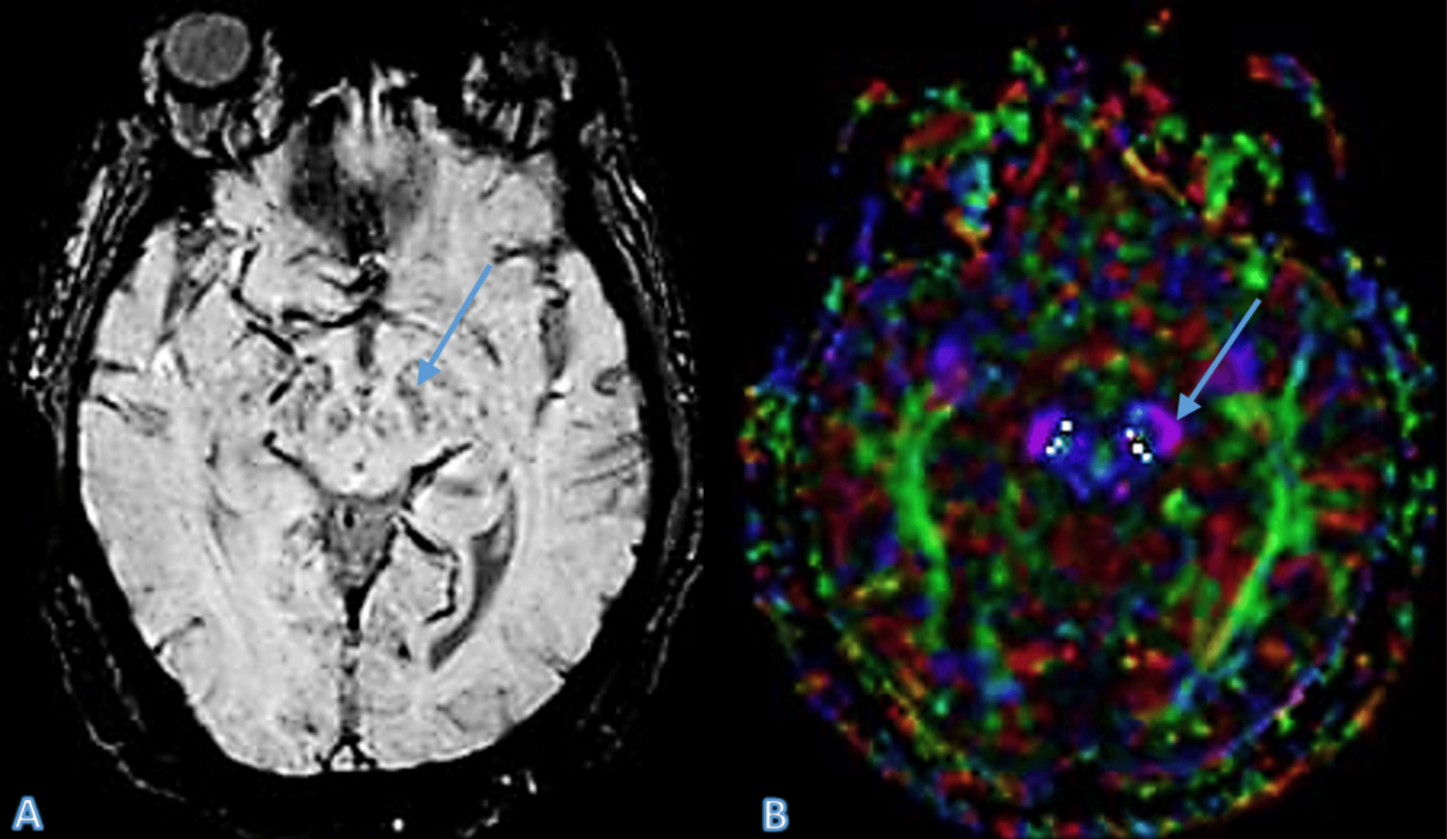
The SWI image (A) shows a normal swallow tail sign, not typically associated with Parkinson’s disease. However, the DTI image (B) reveals a reduction in FA values, which is suggestive of early degenerative changes in the substantia nigra (indicated by arrow), even before these changes are visible on SWI, thereby indicating the sensitivity of DTI in detecting microstructural abnormalities. Arrows in both images highlight the key areas of interest. SWI: Susceptibility-weighted imaging; DTI: Diffusion tensor imaging; FA: Fractional anisotropy

A 72-year-old male patient, presenting with early signs of parkinsonism, underwent an MRI, which initially appeared normal on SWI. However, DTI revealed a mild reduction in FA values in the substantia nigra, indicative of early PD. This case highlights the sensitivity of DTI in detecting microstructural changes that are not yet apparent on conventional MRI (Figure 2).

Case 3

**Figure 3:**
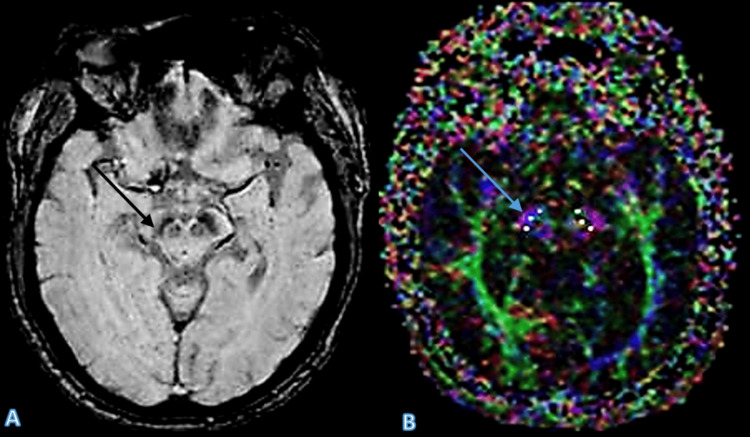
The SWI image (A) demonstrates the absence of the swallow tail sign, which is a hallmark feature of Parkinson’s disease (indicated by the arrow). The DTI image (B) further confirms this diagnosis, showing a pronounced decrease in FA values in the substantia nigra, reflecting the underlying microstructural damage. Arrows in both images highlight the key areas of interest. SWI: Susceptibility-weighted imaging; DTI: Diffusion tensor imaging; FA: Fractional anisotropy

A 65-year-old male patient presented with clinical symptoms consistent with PD. The absence of the swallow tail sign on SWI and the significant reduction in FA values on DTI both supported the diagnosis. This case underscores the utility of combining SWI and DTI for a more comprehensive assessment of PD (Figure 3).

Case 4

**Figure 4:**
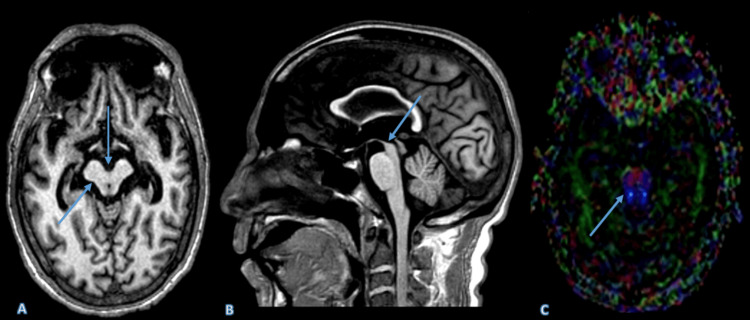
The axial MRI image (A) reveals a concave dorsolateral margin of the midbrain, which is typically convex. The sagittal section (B) emphasizes the pronounced atrophy of the midbrain, with relatively preserved volume of the pons. (C) The DTI analysis further corroborates the diagnosis of progressive supranuclear palsy. (A) The abnormal morphology is highlighted by the presence of a deep interpeduncular cistern, contributing to what is described as the "Mickey Mouse appearance" of the midbrain, as indicated by the arrow. These findings indicate significant midbrain structural changes. (B) This disproportionate atrophy creates the characteristic "hummingbird" or "penguin" sign, where the midbrain resembles the head and beak of a hummingbird or penguin, respectively, as indicated by the arrow. This sign is a hallmark imaging feature of progressive supranuclear palsy and underscores the severity of midbrain degeneration. (C) The DTI gives the diagnosis of progressive supranuclear palsy. The image demonstrates a significant reduction in FA values within the superior cerebellar peduncle, as indicated by the arrow. This reduction in FA values reflects the underlying microstructural damage and loss of white matter integrity in this region, which is characteristic of progressive supranuclear palsy. DTI further confirms the diagnosis by quantifying neurodegeneration that may not be evident on conventional MRI. DTI: Diffusion tensor imaging; FA: Fractional anisotropy

A 70-year-old male with a clinical diagnosis of parkinsonism underwent a conventional MRI, which initially raised suspicion for PSP. The patient presented with decreased cognition, supranuclear vertical gaze palsy (abnormal eye movements), postural instability leading to frequent falls, along with parkinsonian features such as bradykinesia, rigidity, and speech disturbances. Imaging revealed a concave dorsolateral midbrain margin and a deep interpeduncular cistern, producing a "Mickey Mouse appearance" on the axial section, and the midline sagittal image demonstrated significant atrophy of the midbrain, which was concave at its cranial margin producing a "hummingbird" sign. This concavity was also evident laterally on axial images ("morning glory" sign). Further evaluation with DTI confirmed the diagnosis of PSP by demonstrating a marked reduction in FA values in the superior cerebellar peduncle (Figure 4).

Case 5

**Figure 5:**
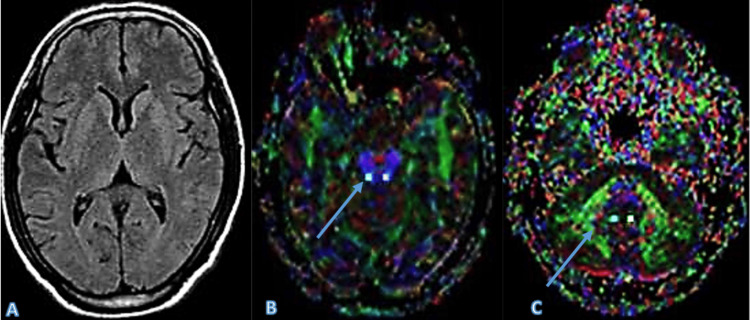
The T2-weighted image (A) appears normal with no obvious abnormalities. The DTI images (B, C), however, show a reduction in FA values in the superior cerebellar peduncle and dentate nucleus, which are regions implicated in essential tremor, providing critical diagnostic information that conventional MRI could not. Arrows in the DTI images indicate areas of FA reduction. DTI: Diffusion tensor imaging; FA: Fractional anisotropy

A 76-year-old male patient, diagnosed with ET, displayed normal findings on conventional MRI. However, DTI revealed a reduction in FA values in the superior cerebellar peduncle and dentate nucleus, which is characteristic of ET. This case demonstrates the importance of DTI in differentiating ET from other movement disorders when conventional imaging appears normal (Figure 5).

MRI findings

Distribution of classical conventional MRI findings in a cohort of 30 patients. Of these, 14 cases (46.7%) were classified as normal, 12 cases (40%) were identified as having PD, and four cases (13.3%) as having PSP. This distribution highlights the conventional MRI findings' ability to differentiate among these neurological conditions albeit with limitations when compared to advanced imaging techniques like DTI (Table 3).

**Table 3:** Distribution of conventional MRI findings and their frequency in Parkinson's disease, progressive supranuclear palsy, and normal cases

Conventional MRI Findings	Frequency	Percentage (%)
Normal	14	46.7
Parkinson's disease	12	40.0
Progressive supranuclear palsy	4	13.3
Total	30	100

Among 30 cases, MRI results showed 14 normal, 12 PD, and four PSP in correlation with clinical diagnosis (Table 4).

**Table 4:** Comparison between clinical diagnosis and MRI results

Clinical Diagnosis	Conventional MRI Results			
	Normal	Parkinson's disease	Progressive supranuclear palsy	Total
Parkinson's disease	3	6	2	11
Essential tremor	5	0	0	5
Atypical parkinsonian disorder	1	3	1	5
Progressive supranuclear palsy	2	0	1	3
Drug-induced parkinsonism	1	1	0	2
Vascular parkinsonism	1	1	0	2
Dementia	1	1	0	2
Total	14	12	4	30

DTI findings

DTI results of 30 patients diagnosed with either PD or APS. Out of the 30 patients, 25 cases (83.3%) showed positive findings on DTI, indicating abnormalities consistent with neurodegenerative disorders. Specifically, PD was identified in 15 patients (50%), ET in four patients (13.3%), and PSP in six patients (20%). Only five patients (16.7%) had normal DTI results, highlighting the effectiveness of DTI in detecting abnormalities associated with these neurodegenerative disorders (Table 5).

**Table 5:** DTI results in patients with Parkinson’s disease, atypical parkinsonian syndromes, and progressive supranuclear palsy DTI: Diffusion tensor imaging

DTI Findings	Number of Cases	Percentage (%)
Positive findings	25	83.3%
Negative findings (Normal DTI)	5	16.7%
Breakdown of positive findings		
Parkinson’s disease	15	50.0%
Essential tremor	4	13.3%
Progressive supranuclear palsy	6	20.0%

Among 30 cases, DTI results showed five normal, 15 PD, four ET, and two PSP cases in correlation with clinical diagnosis (Table 6).

**Table 6:** Correlation between clinical diagnosis with DTI findings DTI: Diffusion tensor imaging

Clinical Diagnosis	DTI Findings			
	Normal	Parkinson's disease	Essential tremor	Progressive supranuclear palsy
Parkinson's disease	2	7	0	2
Essential tremor	2	0	3	0
Atypical parkinsonian syndromes	0	3	1	1
Progressive supranuclear palsy	0	0	0	3
Drug-induced parkinsonism	0	2	0	0
Vascular parkinsonism	1	1	0	0
Dementia	0	2	0	0
Total	5	15	4	6

Correlation between MRI and DTI findings

The correlation between conventional MRI and DTI findings in the diagnosis of parkinsonian syndromes revealed significant differences in the detection capabilities of the two modalities. Conventional MRI identified 14 cases as normal, whereas DTI reduced this number to five, demonstrating its enhanced sensitivity in detecting abnormalities. In cases diagnosed with PD, DTI identified 15 cases compared to 12 by conventional MRI, showing a higher diagnostic yield. Notably, DTI detected four cases of ET, a diagnosis that was entirely missed by conventional MRI. Furthermore, DTI identified six cases of PSP compared to four cases detected by conventional MRI. The statistical analysis yielded a p-value of 0.04, indicating that the differences observed between the two imaging modalities were statistically significant. This suggests that DTI provides superior diagnostic accuracy in distinguishing PD and its atypical variants compared to conventional MRI. The findings underscore the importance of integrating DTI into routine clinical practice for the early and accurate diagnosis of parkinsonian syndromes as it enhances the detection of subtle neurodegenerative changes that conventional MRI might overlook (Table 7).

**Table 7:** Correlation between conventional MRI and DTI findings DTI: Diffusion tensor imaging

Diagnosis	Conventional MRI	DTI	P-value
Normal	14	5	0.04
Parkinson's disease	12	15	
Essential tremor	0	4	
Progressive supranuclear palsy	4	6	
Total	30	30	

This study demonstrates the clear superiority of DTI over conventional MRI in accurately differentiating PD from APS and related disorders. DTI achieved a sensitivity of 83.3% and a specificity of 100% for PD, effectively distinguishing it from both normal findings and PSP. For ET, DTI showed a sensitivity of 100% and a specificity of 89.3%, and for APD, it maintained a sensitivity of 100% and a specificity of 86.2%. The performance of DTI in PSP cases was flawless, with both sensitivity and specificity at 100%, underscoring its effectiveness in diagnosing conditions that overlap clinically and radiologically with PD. Additionally, DTI correctly identified dementia-related, drug-induced, and vascular parkinsonism cases, consistently achieving a specificity of 100%. DTI's ability to detect microstructural abnormalities with high sensitivity and specificity establishes it as an essential imaging modality for early and accurate diagnosis of parkinsonian syndromes (Table 8).

**Table 8:** Case-wise distribution and diagnostic performance of DTI DTI: Diffusion tensor imaging; PD: Parkinson’s disease; ET: Essential tremor; PSP: Progressive supranuclear palsy

Clinical Diagnosis	MRI Findings	DTI Findings	Number of Cases	Sensitivity (%)	Specificity (%)
Parkinson’s disease	6 PD; 2 PSP; 3 Normal	7 PD; 2 Normal; 2 PSP	11	83.3	100
Essential tremor	5 Normal	2 Normal; 3 ET	5	100	89.3
Atypical parkinsonian disorder	3 PD; 1 PSP; 1 Normal	3 PD; 1 PSP; 1 ET	5	100	86.2
Progressive supranuclear palsy	2 Normal; 1 PSP	3 PSP	3	100	100
Dementia	1 Normal; 1 PD	2 PD	2	-	100
Drug-induced parkinsonism	1 Normal; 1 PD	2 PD	2	-	100
Vascular parkinsonism	1 Normal; 1 PD	1 Normal; 1 PD	2	-	100

FA values

FA values in various brain regions for patients with PD and APS. The caudal region of the substantia nigra exhibited a mean FA value of 0.547, with 50% of the cases showing a reduction in FA values, indicating its significance in diagnosing PD. The superior cerebellar peduncle showed a mean FA value of 0.697, with 20% of cases displaying FA reduction, while the middle cerebellar peduncle and transverse pontine fibers both showed no FA reductions. The dentate nucleus had a mean FA value of 0.384, with no cases showing FA reduction independently, though 13.3% of cases had reductions in both the superior cerebellar peduncle and dentate nucleus combined. Notably, 16.7% of cases showed no FA reduction across the evaluated brain regions. The reliability of FA measurements was confirmed by ICC analysis, with ICC values exceeding 0.80 for all measured regions, indicating excellent agreement between measurements. This analysis underscores the importance of FA value measurements in specific brain regions for differentiating between PD and APS (Table 9).

**Table 9:** FA values and reduction sites in Parkinson's disease and atypical parkinsonian syndromes FA: Fractional anisotropy

Region of Interest	Mean FA Value	Median FA Value	Standard Deviation	Number of Cases with FA Reduction	Percentage of Cases with FA Reduction
Substantia nigra (Caudal region)	0.547	0.575	0.132	15	50.0%
Superior cerebellar peduncle	0.697	0.730	0.103	6	20.0%
Middle cerebellar peduncle	0.771	0.770	0.039	0	0.0%
Transverse pontine fibers	0.690	0.690	0.037	0	0.0%
Dentate nucleus	0.384	0.385	0.072	0	0.0%
Superior cerebellar peduncle and dentate nucleus combined	-	-	-	4	13.3%
No sites of FA reduction	-	-	-	5	16.7%

The comparison of mean FA values across different brain regions between MRI and DTI diagnoses revealed several key inferences. Both MRI and DTI diagnoses for normal cases show similar FA values, indicating consistency between the two methods in identifying normal brain structure. For PD, both MRI and DTI demonstrate a reduction in FA values in the substantia nigra, which is characteristic of the disease. However, DTI tends to show slightly lower FA values in the substantia nigra and other regions, suggesting that DTI may be more sensitive in detecting the microstructural changes associated with PD. In the case of PSP, both imaging modalities reveal reduced FA values in the superior cerebellar peduncle, which is consistent with the diagnosis. DTI, again, tends to show slightly lower FA values, indicating its potential higher sensitivity. For ET, DTI identifies reduced FA values in the superior cerebellar peduncle and dentate nucleus, regions that conventional MRI does not distinctly highlight, suggesting that DTI may be more effective in differentiating ET from other conditions. Overall, DTI shows a consistent diagnosis pattern of slightly lower FA values in regions affected by neurodegenerative diseases compared to conventional MRI, which suggests that DTI may be more sensitive in detecting early microstructural changes that are not as apparent on conventional MRI. The differentiation of PD and PSP based on FA values in specific brain regions, such as the substantia nigra and superior cerebellar peduncle, further reinforces the utility of DTI as a diagnostic tool, particularly in distinguishing between PD, PSP, and ET. Therefore, the findings suggest that DTI holds promise as a more sensitive imaging technique compared to conventional MRI for differentiating between various parkinsonian syndromes, potentially leading to earlier and more accurate diagnoses (Tables 10-11).

**Table 10:** Mean FA values in various brain regions with MRI diagnosis FA: Fractional anisotropy

MRI Diagnosis	FA Value in Substantia Nigra (Caudal)	FA Value in Superior Cerebellar Peduncle	FA Value in Middle Cerebellar Peduncle	FA Value in Transverse Pontine Fibers	FA Value in Dentate Nucleus
Normal	0.601	0.698	0.778	0.691	0.363
Parkinson's disease	0.446	0.740	0.780	0.686	0.398
Progressive supranuclear palsy	0.662	0.515	0.717	0.697	0.415

**Table 11:** Mean FA values across different brain regions with DTI diagnosis FA: Fractional anisotropy; DTI: Diffusion tensor imaging

DTI Diagnosis	Substantia Nigra (Caudal)	Superior Cerebellar Peduncle	Middle Cerebellar Peduncle	Transverse Pontine Fibers	Dentate Nucleus
Normal	0.634	0.778	0.796	0.702	0.378
Parkinson’s disease	0.440	0.750	0.776	0.673	0.402
Essential tremor	0.670	0.662	0.770	0.715	0.265
Progressive supranuclear palsy	0.661	0.523	0.740	0.706	0.425

## Discussion

The diagnosis and differentiation of PD from APS remains complex due to overlapping symptoms and similar neurodegenerative processes [8]. Traditional diagnostic methods, particularly clinical assessments and conventional MRI, often struggle to detect the subtle microstructural changes that distinguish these conditions, especially in their early stages. This study addresses these challenges by evaluating the diagnostic accuracy of DTI, an advanced MRI technique that provides detailed insights into the integrity of white matter tracts, offering potentially superior sensitivity and specificity in differentiating PD from APS [9].

PD, as noted by Tolosa et al. (2021), is the second most common neurodegenerative disorder, with its prevalence expected to double in the coming decades [10]. The accurate diagnosis of PD is increasingly supported by advancements in biomarkers and imaging techniques, which are essential for the early identification and characterization of the disease. Despite these advancements, the early stages of PD remain difficult to diagnose, making studies like ours crucial for developing and validating more precise diagnostic tools. Our findings align with the literature, particularly the work of Pagano et al. (2016) who emphasized the utility of imaging in increasing the accuracy of differential diagnoses in parkinsonian syndromes [11]. The study demonstrated that DTI provided superior sensitivity and specificity compared to conventional MRI, particularly in detecting early microstructural changes in the substantia nigra and other critical brain regions. The mean FA values in the substantia nigra of PD patients were significantly reduced (mean FA: 0.440), a finding that corroborates the known degeneration of dopaminergic neurons in this region, which conventional MRI often fails to detect at early stages.

The importance of imaging biomarkers in PD, as highlighted by Ryman et al. (2020), is further supported by our results [12]. Our study revealed that DTI could capture early changes within the nigrostriatal system, which are critical for the motor symptoms of PD. These findings are consistent with the broader evidence that iron-sensitive, neuromelanin-sensitive, and diffusion-sensitive MRI measures have high specificity and sensitivity in distinguishing PD from controls. However, the inconsistent ability of these measures to differentiate PD from APS, as noted by Ryman et al., underscores the significance of our study in demonstrating DTI's superiority in this context [12]. Moreover, the study's findings on PSP align with the observations by Shen et al. (2022) regarding the role of the glymphatic system in PD progression [13]. DTI revealed reduced FA values in the superior cerebellar peduncle (mean FA: 0.523), a region implicated in PSP pathology. The correlation between decreased DTI along the perivascular space (DTI-ALPS) and disease severity in PD, as discussed by Shen et al., supports our conclusion that DTI is a valuable tool for monitoring disease progression and identifying early-stage PD, particularly when conventional MRI results are inconclusive.

The multi-modality approach discussed by Tuite et al. (2013) further underscores the potential of DTI in characterizing disease phenotypes and tracking progression [14,15]. By integrating DTI with other advanced imaging methods, such as iron-sensitive sequences and resting-state functional MRI, it may be possible to develop a more comprehensive understanding of the pathophysiology of PD and APS. Our study contributes to this growing body of evidence by demonstrating that DTI not only improves diagnostic accuracy but also has the potential to become a key component of a multi-modal imaging approach for parkinsonian disorders. Additionally, the study’s focus on early diagnosis is supported by Zhang et al. (2015) who demonstrated that diffusion kurtosis imaging (DKI) of the substantia nigra is a sensitive method for early PD diagnosis [16]. The significant reduction in FA values observed in our study parallels the findings of increased mean kurtosis in early PD patients, further validating DTI as a critical tool for early detection and severity evaluation. This study provides robust evidence supporting the integration of DTI into routine diagnostic protocols for parkinsonian disorders [17,18]. By offering superior sensitivity and specificity in differentiating PD from APS and detecting early microstructural changes, DTI stands out as an invaluable tool in clinical practice [19]. Future research should explore the full potential of DTI in conjunction with other advanced imaging modalities to enhance the accuracy and timeliness of PD and APS diagnoses, ultimately improving patient outcomes and informing the development of novel therapeutic strategies [20,21].

Limitations

This study, while demonstrating the superior diagnostic accuracy of DTI over conventional MRI in differentiating PD from APS, is not without limitations. The small sample size of 30 patients restricts the generalizability of the findings, and the retrospective nature of the study introduces potential biases such as selection and recall bias. Conducted in a single center, the study's applicability to broader clinical settings is limited, and the absence of longitudinal follow-up hinders the assessment of disease progression and long-term diagnostic accuracy. Additionally, while DTI offers valuable insights into microstructural changes, it is susceptible to motion artifacts and requires high-quality imaging, making it challenging to implement consistently in all clinical environments. The lack of pathological confirmation as the gold standard for diagnosis further limits the study's conclusions, as the accuracy of the clinical diagnoses used may have influenced the outcomes. Future research should address these limitations by including larger, multi-center, prospective studies with longitudinal follow-up and a broader range of imaging techniques to fully realize DTI's potential in clinical practice.

## Conclusions

This study underscores the superior diagnostic potential of DTI over conventional MRI in differentiating PD from APS. By focusing on specific brain regions like the substantia nigra and cerebellar peduncles, DTI effectively detected subtle microstructural changes, providing higher sensitivity and specificity in diagnosing PD. Given the high sensitivity (95.8%) and specificity (93.8%) demonstrated in this study, DTI should be routinely included in the MRI protocols for patients suspected of having parkinsonian disorders. DTI allows for the precise calculation of FA values in key brain regions, which are critical for accurate diagnosis. Specifically, reduced FA values in the caudal region of the substantia nigra are indicative of PD. Reductions in the middle cerebellar peduncle and transverse pontine fibers suggest multiple system atrophy of the cerebellar type (MSA-C), while reductions in the middle cerebellar peduncle alone point to multiple system atrophy of the parkinsonian type (MSA-P). PSP is identified by FA reductions in the superior cerebellar peduncle, and ET is associated with reduced FA values in both the superior cerebellar peduncle and dentate nucleus. These results support DTI as a reliable biomarker for disease progression and treatment evaluation, highlighting its effectiveness for early diagnosis and timely intervention in Parkinsonian disorders. The findings strongly advocate for integrating DTI into routine clinical practice for comprehensive evaluation of patients with parkinsonian syndromes.
